# Navigating the Clinical Challenges of a Pregnancy-Related Lobular Capillary Hemangioma of the Breast

**DOI:** 10.7759/cureus.78039

**Published:** 2025-01-26

**Authors:** Willem Van Hoecke, Margot Van Daele, Wiebren A Tjalma, Ali Ramadhan

**Affiliations:** 1 Department of Obstetrics and Gynecology, Antwerp University, Antwerpen, BEL; 2 Faculty of Medicine and Health Sciences, Antwerp University, Wilrijk, BEL; 3 Multidisciplinary Breast Clinic, Gynecological Oncology Unit, Department of Obstetrics and Gynecology, Antwerp University Hospital, Edegem, BEL; 4 Department of Pathology, Antwerp University Hospital, Edegem, BEL

**Keywords:** angiogenesis, cancer, clinical, lobular capillary hemangioma, pregnancy, pyogenic granuloma

## Abstract

Rapidly growing lesions always require careful examination. Due to pregnancy, certain pre-existing lesions may become visible or symptomatic due to accelerated growth and present a challenging differential diagnosis. It is essential to investigate these lesions carefully to establish an accurate diagnosis and ensure appropriate treatment. Due to hormonal and physiological changes during pregnancy, cutaneous lesions may arise and develop more rapidly than usual.

In this case report we discuss a lobular capillary hemangioma (LCH) on the left breast that developed rapidly during pregnancy. The management of fast-growing lesions with possible differential diagnoses, especially during pregnancy, is explored. Early recognition and appropriate management of these lesions are crucial because some of them can be life-threatening.

## Introduction

Over the past century, pyogenic granuloma (PG) has been known by various names. In 1897, Poncet and Dor described it as "botryomycosis humaine" [1]. In 1974, Hartzell coined the terms "granuloma pyogenicum" and "granuloma teleangiectaticum" [2]. Later, in 1982, Cooper and Mills introduced the term "lobular capillary hemangioma" (LCH) to describe a group of hemangiomas--polypoid, intradermal, subcutaneous, and intravenous--that share identical microscopic characteristics [3-5].

When LCH develops during pregnancy, it is referred to as granuloma gravidarum, epulides gravidarum, or colloquially as a "pregnancy tumor." Despite these different names, histopathologically, they are identical to non-pregnancy-associated cases [6]. These lesions arise from mucous membranes and skin, with a preference for intraoral or perioral areas during pregnancy. Other commonly described locations include the head, neck, and hands.

LCH typically presents as a rapidly growing vascular tumor or nodule on the skin or mucosa, characterized by a friable surface that bleeds easily. Clinical diagnosis can be challenging, particularly in cases complicated by bleeding, infection, or ulceration. While macroscopic examination may suggest LCH, a biopsy is crucial to rule out malignancy and confirm the diagnosis. The etiology and pathogenesis of LCH remain unclear; however, it is believed to result from local angiogenesis stimulated by an increase in angiogenic factors such as vascular endothelial growth factor (VEGF) and basic fibroblast growth factor (bFGF) [7]. The systemic increase in hormones during pregnancy is also a known trigger. Hormonal changes during pregnancy, particularly in the second or third trimesters, significantly increase the incidence of LCH, affecting up to 5% of pregnant women [8].

This case report describes a 23-year-old pregnant patient who developed a 14 mm lesion on her left breast within one week. While differential diagnoses may include benign lesions, such as cherry hemangiomas, the lesion’s rapid growth and invasive nature warrant consideration of malignancy.

The aim of this case report is to explore the differential diagnosis and management approach for fast-growing lesions during pregnancy.

## Case presentation

**Figure 1:**
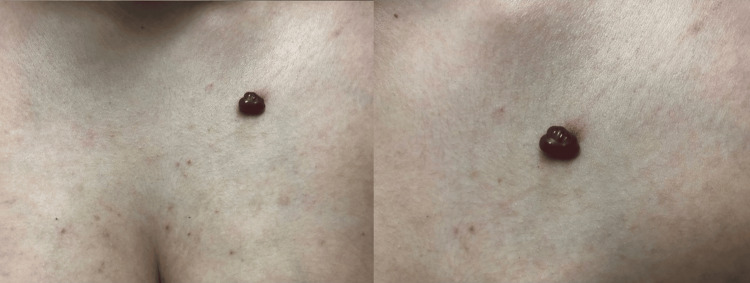
On the left, macroscopically the lesion measured one centimeter in diameter, exhibiting a polypoid, elevated structure with a raspberry-red colour. On the right, a detailed closer view of the lesion is presented.

A 23-year-old woman, G4P3A1, presented at 33 weeks of pregnancy with a painless mass on her left breast. The lesion had appeared one week prior and had been growing rapidly. The mass began bleeding after her son accidentally struck his head against her left breast, prompting the patient to seek medical attention. Upon examination, the lesion measured approximately one centimeter in diameter and displayed a polypoid, elevated structure with a characteristic raspberry-red color (Figure 1).

Upon gentle palpation, immediate bleeding was observed from the lesion due to its fragile tissue. Aside from spontaneous bleeding or bleeding triggered by contact, the patient reported no other systemic symptoms. Upon further examination, this was the only lesion present on her body. Clinical evaluation of the breasts, in line with her gestational age, was unremarkable, with no evidence of axillary or inguinal lymphadenopathy.

Given its rapid development and vascular nature, the lesion was highly suggestive of LCH. However, other benign and malignant possibilities could not be ruled out. To establish a definitive diagnosis, a biopsy was deemed essential. Due to the lesion's high risk of bleeding, an excisional biopsy was preferred. The differential diagnosis prompted a discussion of various treatment options, including ablation, cryotherapy, laser therapy, and surgical excision.

**Figure 2:**
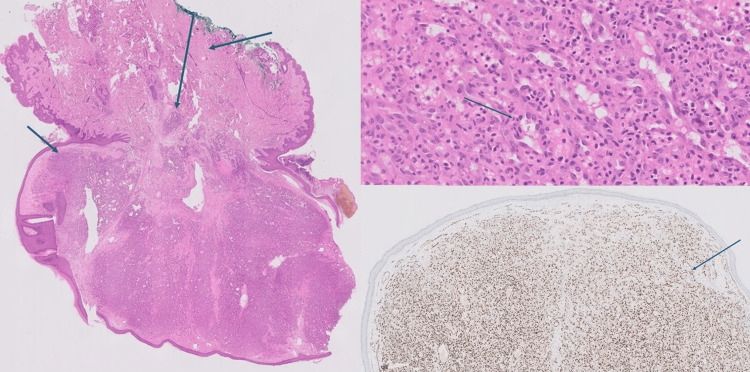
(Left) H&E staining reveals centrally raised pink- to dark-pink lesion is seen with a smooth surface, measuring 14 x 9 mm with closest surgical margins of 2mm, with the excision margins inked green (Top right). H&E staining (detail) : Centrally the dermis contained a prominent branching capillary network resembling vascular lobules, with minimal erythrocytes visible and notable mitotic activity (Bottom right). IHC stain for ERG: The specimen contained a prominent branching capillary network resembling vascular lobules, with minimal erythrocytes visible. H&E: hematoxylin and eosin; IHC: immunohistochemical. Transcriptional regulator ERG is a protein encoded by ERG (ETS family transcription factor ERG), which is located at 21q22. It is a highly sensitive immunohistochemical marker for, among other things, vascular differentiation, immature myeloid differentiation, and cartilaginous differentiation.

Surgical resection was performed under local anesthesia using 2% xylocaine, with the goal of obtaining a definitive histopathological diagnosis. The lesion was completely excised with a macroscopic margin of 5 mm. Histopathology revealed a specimen consisting of a non-oriented elliptical skin excision from the left breast, measuring 14 x 7 mm. At its center, the lesion exhibited a raised pink to dark pink appearance with a smooth surface, measuring 14 x 9 mm and 5 mm in height. The lesion was located 2 mm from the nearest surgical margin, with the excision margins marked in green ink (Figure 2).

Sectioning of the specimen revealed dense pink tissue with a slightly mucinous appearance. Microscopically, the lesion exhibited a mushroom-shaped, raised, partially polypoid structure with overlying atrophic epidermis and peripheral acanthosis. At the center, the dermis displayed a prominent branching capillary network, resembling vascular lobules, with minimal erythrocyte presence and notable mitotic activity (Figure 2). No ulceration, atypia, or evidence of malignancy was observed. The histopathological findings were consistent with an LCH of the skin of the breast.

The patient was discharged on the same day as the excision in good general health, and no recurrence was noted during the remainder of the pregnancy.

## Discussion

A lobular capillary hemangioma is a benign, vascular lesion of the skin and mucosa. Different subtypes of LCH are described with the most frequent being the cutaneous type (86%) and mucosal type (12%) [9]. The precise etiology of LCH is unknown. There are however several predisposing factors for the development of LCH (Table 1) [10,11].

**Table 1:** Predisposing factors for LCH LCH: lobular capillary hemangioma

Predisposing Factors	Description
Trauma	Minor injuries or chronic irritation, especially in the oral cavity
Hormonal changes	Associated with pregnancy, where elevated hormone levels contribute to lesion growth, particularly in the second and third trimesters
Medications	Systemic and topical retinoids
	Antiretrovirals
	- Indinavir (HIV protease inhibitor)
	Antineoplastics
	- Pyrimidine analogs: capecitabineand systemic 5-fluorouracil
	- Taxanes: docetaxel and paclitaxel
	- Epidermal growth factor receptor (EGFR) inhibitors
	° Monoclonal antibodies against the EGFR: cetuximab and panitumumab
	° EGFR tyrosine kinase inhibitors (orally active small molecule): gefitinib, erlotinib, lapatinib, afatinib, and osimertinib
	- Tyrosine kinase inhibitor (imatinib)
	- BRAF inhibitors: vemurafenib, encorafenib
	Immunosuppressive agents
	- Tumor necrosis factor-alpha (TNF-alpha) antagonists: etanercept
	- Mammalian target of rapamycin (mTOR) inhibitors
Vascular malformations	Pre-existing vascular abnormalities
Infections	Bacterial or other infections (like herpes virus type 1 or Orf virus)
Underlying conditions	Sturge-Weber syndrome, chronic dermatoses (atopic dermatitis and psoriasis)

The pathogenesis of LCH is primarily linked to an imbalance between pro-angiogenic and anti-angiogenic factors. The MAPK pathway may be attributed to the physiologic and pathologic angiogenesis. Staining for the phosphorylated form of MAPK (P-MAPK) may help the pathologist differentiate benign from malignant tumors. Inhibitors of the MAPK pathway may be useful for treatment options. Trauma or irritation (such as infection) is associated with the development of approximately 7-50% of LCH cases, as seen in our patient [10,12,13]. During pregnancy, the immune response is heightened due to increased levels of sex hormones like estrogen, further contributing to the lesion's development. Notably, around 30% of periungual LCH cases are associated with the use of certain medications.

Clinically, LCH often presents as a small, shiny red or pink papule [14]. The lesion is typically elevated and may have an irregular surface. It undergoes rapid growth initially before stabilizing. In most cases, it is painless and highly vascularized, making it prone to oozing, bleeding, and ulceration. The ease of bleeding, particularly during pregnancy, is a significant concern for patients and increases susceptibility to infection. When located in the esophagus, LCH can cause difficulty swallowing, and if situated in the airway, it may lead to breathing problems. Rapidly growing cutaneous lesions, especially during pregnancy, present a clinical challenge. It is crucial to accurately identify these lesions, as some may be malignant.

Table 2 presents a list of fast-growing lesions that may occur during pregnancy, along with their clinical presentations and associated risks [3,4,6,7,10]. Given the potential dangers linked to these lesions, accurate diagnosis and appropriate treatment are of paramount importance. For instance, while basal cell carcinoma may remain indolent for an extended period, it can accelerate and ulcerate during pregnancy. Similarly, although melanoma is rare, its prognosis can be significantly worsened if diagnosed too late, highlighting the critical need to distinguish it from conditions like LCH.

**Table 2:** Differential diagnoses of fast-growing cutaneous lesions during pregnancy together with their presentation and risks

Lesion Type	Presentation	Risks
Benign Lesions		
Hemangioma	Bright red (mostly) birthmark; rubbery bump or flat red patch	Ulceration, pain, bleeding, scarring, infection
Peripheral giant cell granuloma	Red to bluish-purple, usually more blue than pyogenic granuloma	Ulceration, infection
Keratoacanthoma	Crateriform appearance, ulcer-like	Scarring, ulceration, bleeding
Angiokeratoma	Dark bluish-red lesion with crust; often multiple and easily bleeding	N/A
Lobular capillary hemangioma	Rapidly growing, easily bleeding, red lesion on skin or mucosa	Bleeding, ulceration, infection
Cherry angioma	Asymptomatic, solitary red lesion growing with age	N/A
Bacillary angiomatosis	Wart-like lesion, black-red, often multiple	Immune deficiency
Verruga perugana	(Solitary) angiomatous nodule	Bartonella bacilliformis infection
Malignant Lesions		
Basal cell carcinoma	Flat, firm, pale area; small, raised, pink or red; easily bleeding	Recurrence, metastasis
Squamous cell carcinoma	Red nodules or crusty patches; often an open wound	Metastasis
Nodular melanoma	Elevated, firm lesion; dark-colored; rapidly growing	Metastasis
Merkel cell tumor	Flesh-colored or bluish-red nodule; fast-growing; painless	Metastasis
Kaposi’s sarcoma	Purple, brown, or red spots or patches; bumpy or smooth	HIV (new diagnosis or exacerbation)
Cutaneous neuroblastoma	Fleshly red nodule on skin; heterogeneous, with multiple bruises; rapidly growing lump under skin	Invasion, metastasis, death

Differentiating LCH from its differential diagnoses based solely on clinical assessment can be particularly challenging. While dermoscopy may provide additional insights, histopathological examination remains essential for a definitive diagnosis, which is crucial for guiding treatment decisions. This is especially important because interventions such as laser resection or cryotherapy can interfere with the diagnostic process; these treatments can destroy tissue, making subsequent pathological analysis impossible and potentially delaying both accurate diagnosis and appropriate treatment.

In this case, resection under local anesthesia was performed as the curative treatment of choice, as it is associated with the lowest recurrence rate and allows for thorough histopathological evaluation (Table 3).

**Table 3:** Treatment options for LCH LCH: lobular capillary hemangioma

Treatment Method	Recurrence Rate
Surgical excision	5-16%
Curettage and electrocautery	10-20%
Laser therapy (Nd, CO₂)	10-15%
Imiquimod cream (5%)	20-30%
Timolol gel (0.5%)	20-25%
Intralesional steroids	25-35%
Cryotherapy	15-20%

Alternative treatment options for LCH include curettage, electrocautery, laser therapy, cryotherapy, local gels such as imiquimod or timolol, and intralesional steroids [15] (Table 3). The choice of treatment depends on factors such as the size, location, and extent of the lesion. Given that LCH is typically a benign condition, a watchful waiting approach may be appropriate in certain cases. During pregnancy, lesion removal is not always necessary, as spontaneous regression is common after childbirth.

In this patient’s case, surgical resection was preferred due to the uncertain nature of the lesion and concerns about potentially missing a more serious diagnosis. This approach ensured both a definitive treatment and the ability to conduct histopathological analysis, allowing for an accurate diagnosis and timely management.

## Conclusions

Several important diagnoses must be considered for a fast-growing lesion on the skin, especially during pregnancy. The diagnosis of LCH or pyogenic granuloma is rare but its changes are increased during pregnancy. The preferred treatment for these lesions is local excision, which effectively minimizes complications like bleeding, ulcerations, infections, or recurrence. Furthermore, this approach provides an accurate pathological diagnosis, ensuring timely detection of any potential malignancies.

Fast-growing lesions during pregnancy are often influenced by hormonal changes, which contribute to increased vascular growth and may lead to the formation of tumors. These pregnancy-related tumors can be either benign or malignant. The rapid growth of these lesions, along with their tendency to bleed easily, can sometimes mimic malignancy, making their appearance confusing and challenging to diagnose. To ensure an accurate diagnosis, it is crucial to obtain a biopsy. This is the only way to rule out malignancies and confirm the true nature of the lesion.
